# Life, death, and statins: association of statin prescriptions and survival in older general practice patients

**DOI:** 10.1017/S1463423624000161

**Published:** 2024-05-16

**Authors:** Adam J Hodgkins, Judy Mullan, Darren J Mayne, Andrew Bonney

**Affiliations:** 1 Graduate School of Medicine, Faculty of Science, Medicine and Health, University of Wollongong, Wollongong, NSW, Australia; 2 Illawarra Health and Medical Research Institute, University of Wollongong, Wollongong, NSW, Australia; 3 Centre for Health Research Illawarra Shoalhaven Population, University of Wollongong, Wollongong, NSW, Australia; 4 Illawarra Shoalhaven Local Health District, Public Health Unit, Warrawong, NSW, Australia; 5 The University of Sydney, Sydney School of Public Health, Camperdown, NSW, Australia

**Keywords:** aged, data collection, deprescriptions, electronic health records, feasibility studies, general practice, humans, hydroxymethylglutaryl-CoA reductase inhibitors, mortality, prescriptions, primary health care, proportional hazards models, retrospective studies, survival analysis

## Abstract

**Aims:**

This study serves as an exemplar to demonstrate the scalability of a research approach using survival analysis applied to general practice electronic health record data from multiple sites. Collection of these data, the subsequent analysis, and the preparation of practice-specific reports were performed using a bespoke distributed data collection and analysis software tool.

**Background:**

Statins are a very commonly prescribed medication, yet there is a paucity of evidence for their benefits in older patients. We examine the relationship between statin prescriptions for general practice patients over 75 and all-cause mortality.

**Methods:**

We carried out a retrospective cohort study using survival analysis applied to data extracted from the electronic health records of five Australian general practices.

**Findings:**

The data from 8025 patients were analysed. The median duration of follow-up was 6.48 years. Overall, 52 015 patient-years of data were examined, and the outcome of death from any cause was measured in 1657 patients (21%), with the remainder being censored. Adjusted all-cause mortality was similar for participants not prescribed statins versus those who were (HR 1.05, 95% CI 0.92–1.20, *P* = 0.46), except for patients with diabetes for whom all-cause mortality was increased (HR = 1.29, 95% CI: 1.00–1.68, *P* = 0.05). In contrast, adjusted all-cause mortality was significantly lower for patients deprescribed statins compared to those who were prescribed statins (HR 0.81, 95% CI 0.70–0.93, *P* < 0.001), including among females (HR = 0.75, 95% CI: 0.61–0.91, *P* < 0.001) and participants treated for secondary prevention (HR = 0.72, 95% CI: 0.60–0.86, *P* < 0.001). This study demonstrated the scalability of a research approach using survival analysis applied to general practice electronic health record data from multiple sites. We found no evidence of increased mortality due to statin-deprescribing decisions in primary care.

## Introduction

There is a relative paucity of research emanating from general practice. (Heal and Roberts, 2019) One consideration in addressing this issue is the potential to use routinely collected computer data for primary care research. (de Lusignan and van Weel, 2006; Benchimol *et al.*, 2015) Using data collected from general practice is an appropriate way to inform clinical care in general practice. Data collected about patients, their health conditions, and their treatments within the environment in which they occur provide important contextual factors which contribute to the applicability of research results. (Stange, Miller and McWhinney, 2001)

Randomised controlled trials are important for informing clinicians of the ability of treatments to make a difference in ideal circumstances (efficacy); however, pragmatic trials are also needed to measure the degree of benefit in a real-world setting (effectiveness). (Porzsolt *et al.*, 2015) Analysis of data collected routinely in the course of providing health care and recorded in the electronic health record (EHR) is well suited to pragmatic trials and comparative effectiveness research. (Cowie *et al.*, 2017; Hurwitz *et al.*, 2017)

One approach to facilitate more longitudinal research originating from the general practice environment is to use survival analysis with data recorded in the EHR. (Virnig *et al.*, 2000; Hodgkins *et al.*, 2018; Putzel *et al.*, 2021) We wish to develop and demonstrate the scalability of a method of performing such research using a bespoke distributed data collection and analysis software tool.

It is important to address potential barriers and enablers in deploying research projects within the general practice environment. The study design aims to address identified concerns of general practitioners (GPs) in relation to using EHR data for research. (Hodgkins *et al.*, 2020) It collects and analyses data without imposing excessive time burdens on GPs or practice staff. It does not disrupt the routine delivery of care. Patient privacy is ensured by only using deidentified data. Analysis and preparation of an automated report are performed within each general practice. This report is able to inform GPs of the outcomes of healthcare delivery in their own practice and may be helpful in guiding future care.

In developing the methods for this project, a study was conducted to address the association of all-cause mortality with prescribing or deprescribing lipid-lowering medication in older general practice patients. (Hodgkins *et al.*, 2019) That study was conducted in a single practice and showed feasibility to generate valuable outcomes. (Hodgkins *et al.*, 2019) The all-cause mortality risk associated with specific factors which were described in that study correlated well with existing research, (Gellert, Schöttker and Brenner, 2012) suggesting that the design was able to identify such risk when it was present. The single-practice study had high internal validity; however, there was limited external validity. In order to generate acceptable generalisability in future research, it is desirable to access a study sample that is representative of the study population of older general practice patients. This study demonstrates the feasibility to scale the single-practice approach to use data from multiple practices.

GPs prefer that research using EHR data is used for study relevant to general practice care. (Hodgkins *et al.*, 2020) This exemplar study examines the association between statin prescribing and deprescribing, and mortality in older general practice patients, a subject very relevant to primary healthcare.

Statin prescriptions are common in Australia. In the year ending June 2021, lipid-lowering medication accounted for over 32 million prescriptions. (*Pharmaceutical Benefits Scheme (PBS) Expenditure and Prescriptions Report 1 July 2020 to 30 June 2021, no date*) In this same year, the top two medications by prescription volume in Australia were rosuvastatin and atorvastatin. These drugs also occupied the top two places on a list of the highest subsidised prescriptions. (*Pharmaceutical Benefits Scheme (PBS) Expenditure and Prescriptions Report 1 July 2020 to 30 June 2021, no date*) The vast majority of statins prescriptions are initiated by GPs (Simons, Ortiz and Calcino, 2011), and almost all repeat prescriptions are supplied in general practice. Such commonly prescribed medications require close examination of their effectiveness, especially in real-world use.

Statin use can improve mortality in some groups. In 1994 the 4S study established that all-cause mortality was decreased in patients aged 35 to 70 with ischaemic heart disease. (Scandinavian Simvastatin Survival Study Group, 1994) The following year the West of Scotland group demonstrated benefit in using statins for primary prevention for men under the age of 65. (Shepherd *et al.*, 1995) The PROSPER study examined the use of statins in older patients, but despite finding reduced incidence of some events, it was unable to demonstrate a benefit in all-cause mortality. (Shepherd *et al.*, 2002)

There is still some doubt about the efficacy of statins to reduce death rates in older patients. (LaRosa, 2014; Zoungas *et al.*, 2014; Teng *et al.*, 2015; Gurwitz, Go and Fortmann, 2016) Much of the available literature suggests that there may be reductions in cardiovascular events when statins are prescribed to older patients as secondary prevention. (Teng *et al.*, 2015) However, the evidence is lacking for primary prevention in this age group and there is a paucity of evidence to suggest that statins influence all-cause mortality. (LaRosa, 2014; Zoungas *et al.*, 2014; Teng *et al.*, 2015; Gurwitz, Go and Fortmann, 2016) More research is needed. (Gurwitz, Go and Fortmann, 2016)

A key aim of this study is to demonstrate a methodology which uses real-world data from multiple practices to produce robust longitudinal research outcomes and at the same time, provide clinicians with feedback at a practice level. The authors are not aware of any other similar published studies.

We investigate the feasibility of scaling our methodology to facilitate practice-based effectiveness research using statin prescribing to older patients in a general practice setting and survival analysis of longitudinal data on all-cause mortality from the EHR as a motivating example. Specifically, we consider:

Do decisions made in clinical practice to cease statin prescriptions in older patients affect mortality?

Is there a difference in mortality between older patients prescribed statins and those who have never had statins prescribed?

## Methods

We carried out a retrospective cohort study using survival analysis applied to data extracted from the Best Practice Software (Pyefinch, 2021) databases of five Australian general practices. The deidentified data were extracted and analysed on site to produce individual practice-level reports which presented outcomes in a similar format to this paper. Selected, deidentified data were forwarded securely to the researchers for the grouped analysis which is described subsequently.

### Inclusion and exclusion

**Figure 1.**
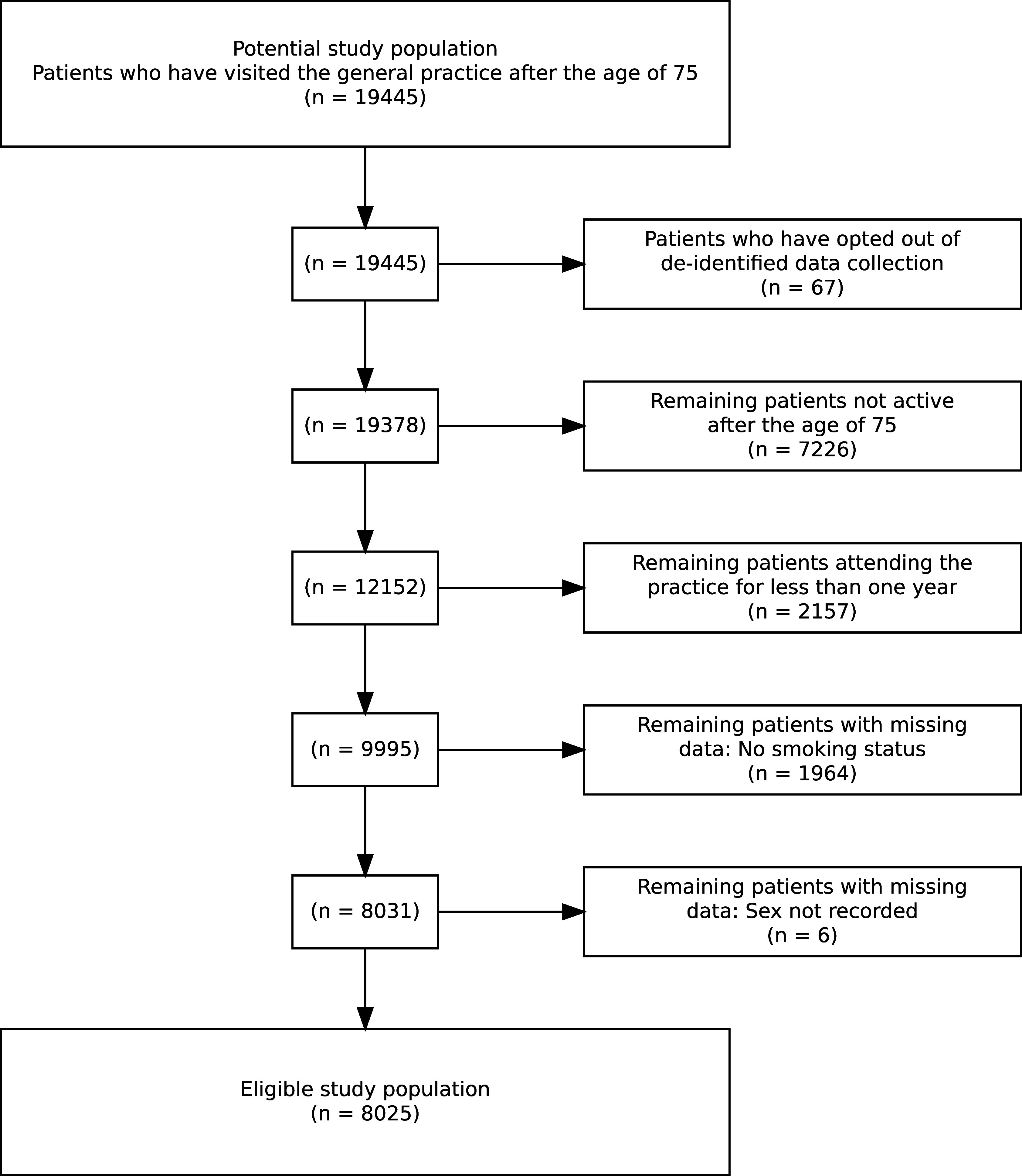
The exclusion criteria applied to the practice data

Patients who had attended one of the participating practices after the age of 75 were eligible for inclusion. Patients who have opted out of deidentified data collection at their practices were excluded from the study population. We excluded patients who were not considered ‘active’ (defined as having a minimum of three visits in the two years prior to study entry). (The Royal Australian College of General Practitioners, 2020) This exclusion was considered appropriate as prescription data was less likely to be accurate for patients not considered ‘active’. We excluded patients for missing data if smoking status or sex were not recorded in the EHR. Figure 1 shows how participants were selected for inclusion in the study.

### Index & endpoints

The index date was the latter of the patient’s 75th birthday, one year after their first practice visit, or the earliest visit when the patient could be defined as an active patient of the practice.

We wished to study the survival of older patients using reliable data from the EHR. If a patient had not been a patient of the practice for at least a year, then prescription data may not reflect the usual medications. Similarly, if a patient was not ‘active’ then there is a possibility that prescriptions obtained elsewhere may be misinterpreted as cessation of the medication.

The study endpoint was patient death from any cause. This was determined by the date of death documented in the electronic health record. If there was no date of death recorded in the EHR, then data were censored at the last visit date when the patient was considered active (that is the last visit date which was preceded by two or more visits within a two-year period).

### Statistical analysis

Patients were classified according to their history of statin prescriptions. ‘Non-users’ had no record of statin prescription. ‘Continuous users’ had been prescribed statins on more than one occasion and the last recorded prescription was less than 12 months prior to death or censoring. ‘Stoppers’ includes those previously prescribed statins but without a prescription for at least a year prior to death or censoring.

The all-cause mortality risks of these three groups of patients were compared. Kaplan-Meier curves were used to estimate survival differences. Hazard ratios (HR) were calculated with adjustment for possible confounders using Cox proportional hazard regression with continuous users as the reference category. Possible confounders which were adjusted for include age at study entry, number of prescriptions for any medication per year (previously demonstrated as a proxy for multimorbidity), (Huntley *et al.*, 2012; Brilleman and Salisbury, 2013) smoking status at the end of the study, residential aged care, and the presence of ischaemic heart disease, cerebrovascular disease, peripheral vascular disease, or diabetes. Practice attended was included as a fixed effect in all analyses to account for clustering in the study design (McNeish and Kelley, 2019). We also planned to undertake sub-group analyses stratified by sex, smoking status, a history of diabetes, and a history of cardiovascular, cerebrovascular, or peripheral vascular disease to determine primary or secondary prevention. Data analysis was performed using R (R Core Team, 2022) with two-tailed significance tests and a type 1 error rate of 0.05. Specifically, the R Survival package (Therneau, 2022) was used for generating survival curves and calculating HR.

## Results

### Characteristics of the study population

The data from 8025 patients from five general practices were analysed. All practices were located in regional New South Wales (NSW) in Modified Monash Model 3 (MMM3) regions. (Versace *et al.*, 2021) The number of eligible patients contributed by each practice ranged from 825 to 2894. The median duration of follow-up for all patients was 6.48 years. Overall, 52 015 patient-years of data were examined, and the outcome of death from any cause was measured in 1657 patients (21%), with the remainder being censored. The age at study entry ranged from 75 to 99 years, with the median of 75.2 years of age. Table 1 shows the characteristics of the eligible patients aged over 75 included in the study.

**Table 1.** Attributes of the study population

	All	Non-user	Continuous user	Stopper
N	8025	3952	2625	1448
Deaths (%)	1657 (20.6)	815 (20.6)	450 (17.1)	392 (27.1)
Men (%)	3454 (43.0)	1621 (41.0)	1282 (48.8)	551 (38.1)
Non-smoker (%)	4318 (53.8)	2239 (56.7)	1349 (51.4)	730 (50.4)
Ex-smoker (%)	3433 (42.8)	1579 (40.0)	1199 (45.7)	655 (45.2)
Smoker (%)	274 (3.4)	134 (3.4)	77 (2.9)	63 (4.4)
Non-statin lipid medication (%)	599 (7.5)	124 (3.1)	287 (10.9)	188 (13.0)
RACF resident	1363 (17.0)	656 (16.6)	233 (8.9)	474 (32.7)
Diabetes (%)	1653 (20.6)	479 (12.1)	801 (30.5)	373 (25.8)
Ischaemic heart disease (%)	2054 (25.6)	546 (13.8)	1001 (38.1)	507 (35.0)
Cerebrovascular disease (%)	1342 (16.7)	473 (12.0)	545 (20.8)	324 (22.4)
Peripheral vascular disease (%)	412 (5.1)	120 (3.0)	168 (6.4)	124 (8.6)
Duration (mean (SD))	6.48 (4.48)	5.99 (4.38)	6.06 (4.03)	8.60 (4.89)
Age at first observation (mean (SD))	77.52 (4.26)	78.50 (4.92)	76.45 (3.03)	76.80 (3.53)
Number of prescribed drugs (mean(SD))	9.78 (6.05)	8.72 (5.90)	11.04 (5.84)	10.36 (6.31)

### Association of statins and survival

**Figure 2.**
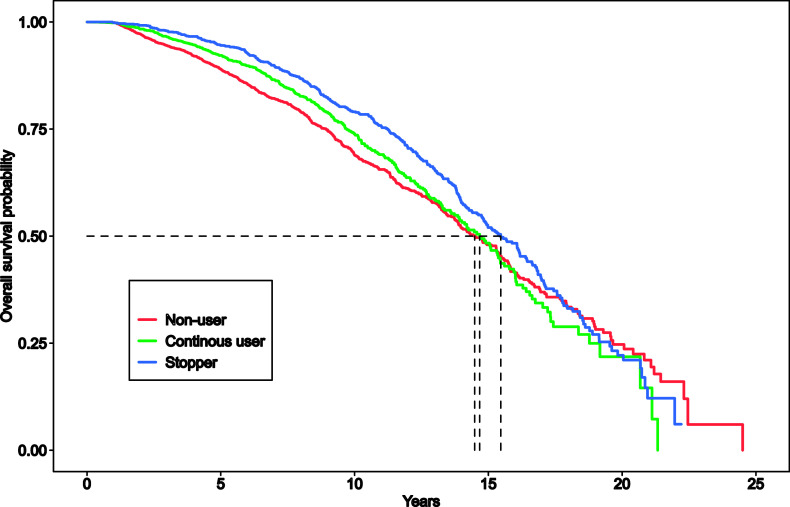
Kaplan-Meier survival curves for different groups of patients according to statin prescriptions

The study population of Australian general practice patients aged over 75 were stratified by their statin prescription status into statin ‘continuous users’, ‘non-users’, and ‘stoppers’. The survival of each group was estimated, and a comparison is shown in the Kaplan-Meier curves in Figure 2. The median survival times for each group with 95% confidence intervals are reported in Table 2.

**Table 2.** Median survival according to statin prescriptions

Exposure	N	Events	Median survival (yrs)	95% CI
Non-user	3952	815	14.49	13.88–15.33
Continuous user	2625	450	14.67	13.91–15.45
Stopper	1448	392	15.47	14.82–16.39

### Adjusted all-cause mortality HRs

**Figure 3.**
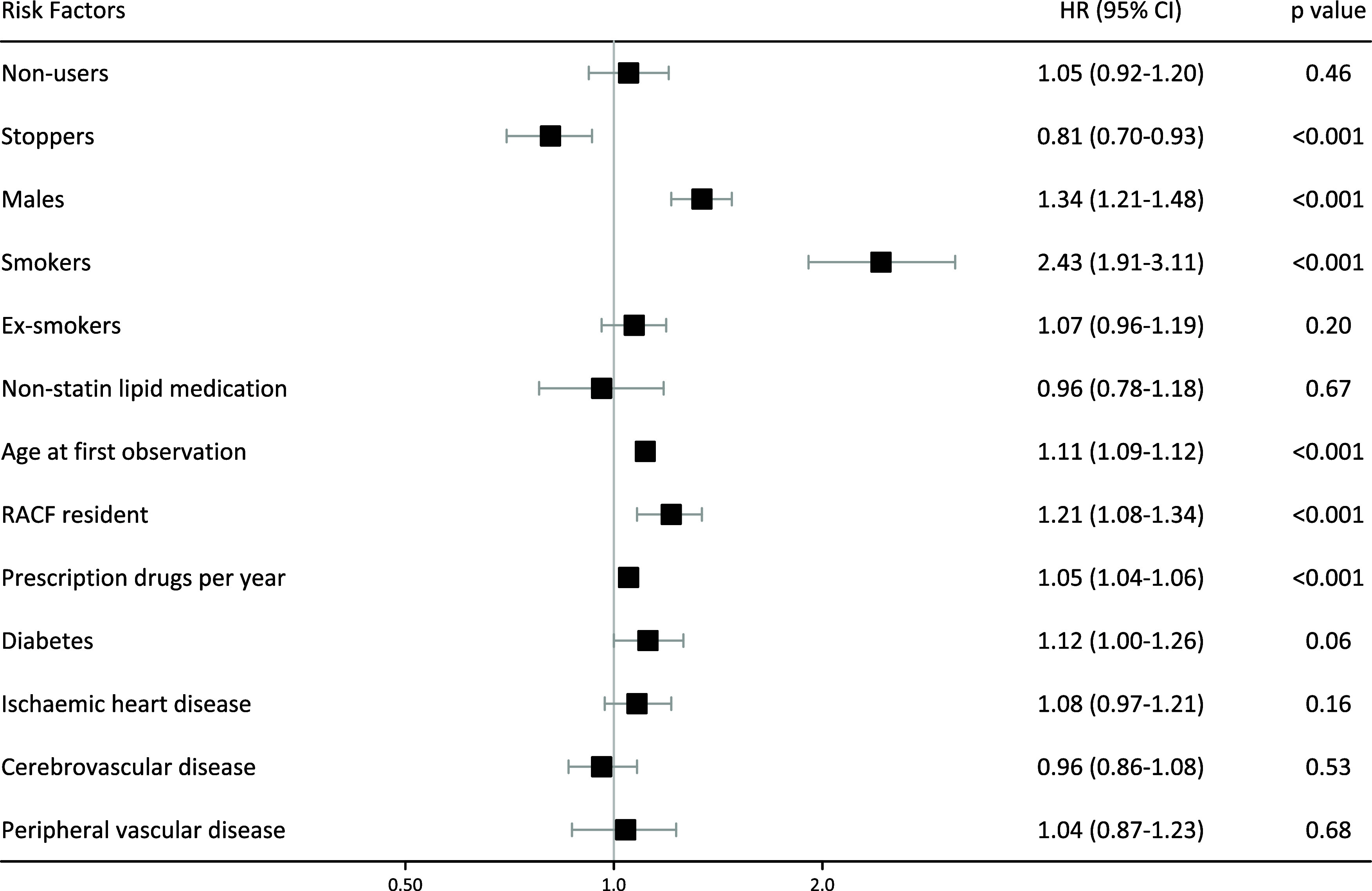
Adjusted all-cause mortality hazard ratios

HR and 95% confidence intervals (95% CI) of all-cause mortality adjusted for all other variables were estimated using Cox regression models and are shown in Figure 3. In fully adjusted Cox models, all-cause mortality was significantly reduced for stoppers but similar for non-users compared to continuous users. The HR of all-cause mortality was also higher in males, smokers, and residents of aged care facilities. The numerical variables of age and multimorbidity (as measured by the proxy of a count of medications prescribed in a year) were both associated with an increased HR.

### Subgroups of patients

**Figure 4.**
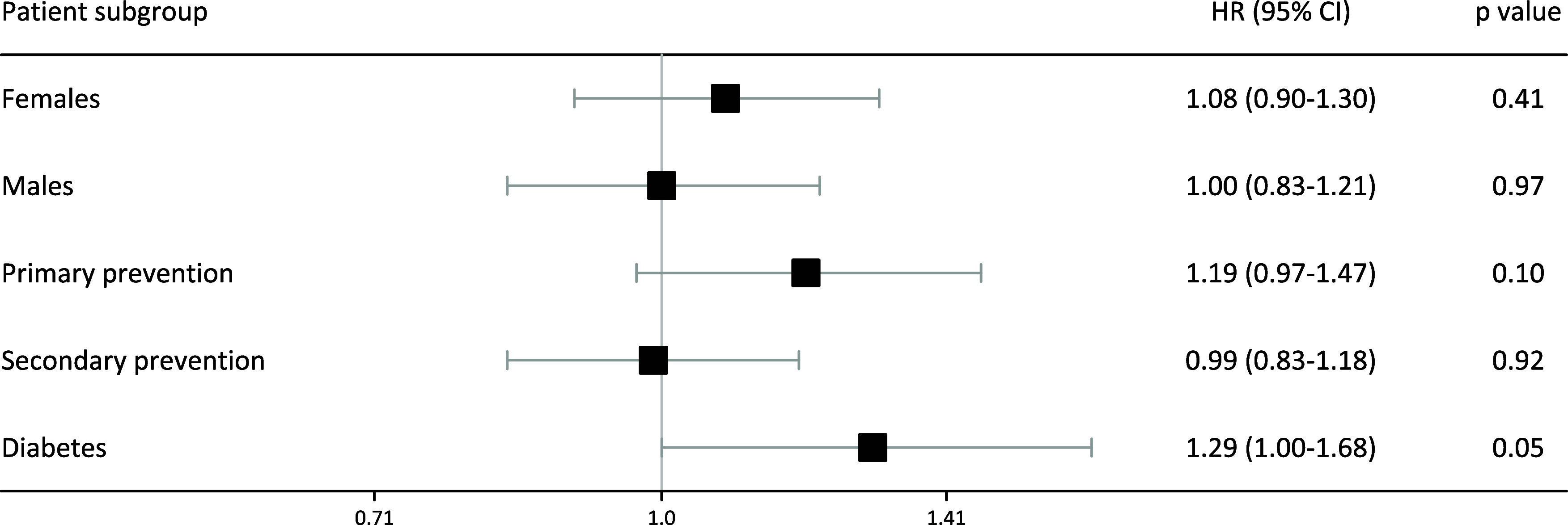
Hazard ratios of statin ‘non-users’ compared with ‘continuous users’ for patient subgroups

It is of interest to know if certain patient groups have a different rate of survival depending on their statin use. We looked at the survival rates in various groups of patients, adjusted the risk for all other confounders, and calculated the HRs of these groups in ‘non-users’ compared to ‘continuous users’. Figure 4 shows these outcomes.

There was a trend towards increased mortality for patients who did not use statins when compared to those who were prescribed these medications. However, this was not statistically significant for most groups. The exception was in diabetics who were not prescribed statins who had a statistically significant higher rate of death compared with patients with ongoing prescriptions for these medications.

**Figure 5.**
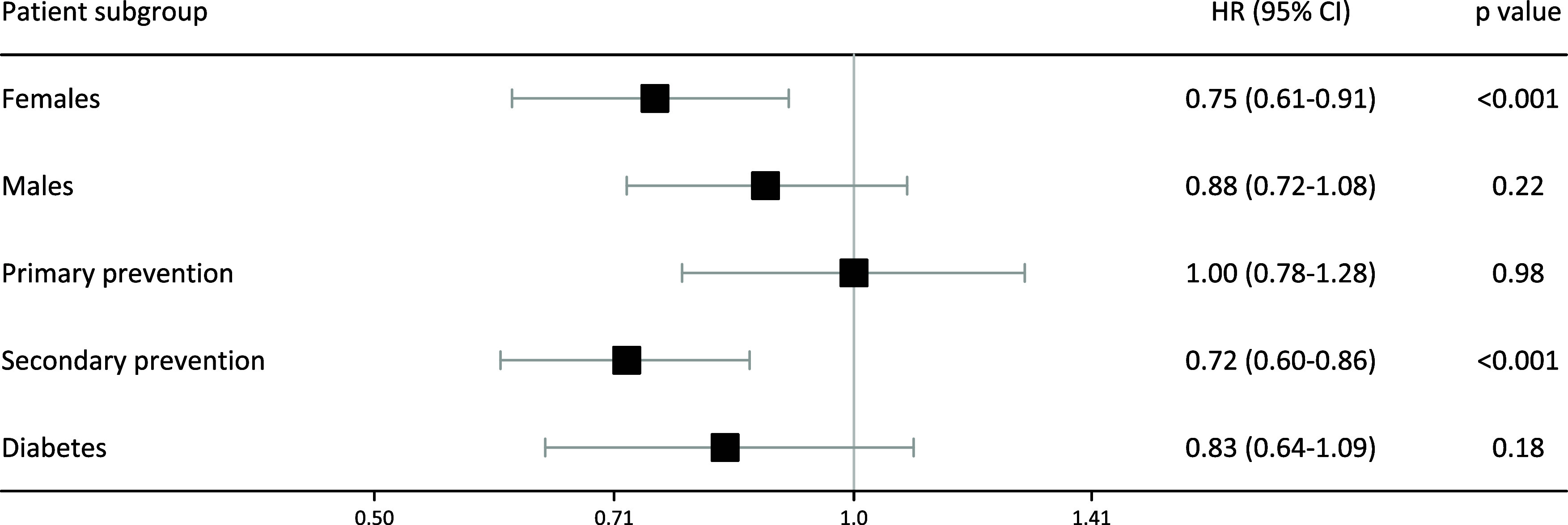
Hazard ratios of statin ‘stoppers’ compared with ‘continuous users’ for patient subgroups

A similar analysis was performed to compare ‘stoppers’ to ‘continuous users’. Figure 5 shows these outcomes. Rates of death among patients who had ceased prescriptions for statins were significantly lower for females and patients treated as secondary prevention. There was no association between stopping statin use and death for males, for patients treated as primary prevention, or for diabetics.

This study also produced individual practice-level reports which replicated the above analysis for each participating practice using their own data. Our ethics approval did not extend to reporting these outputs as they may have the potential to identify individual practices.

## Discussion

The aim of this paper is to demonstrate that important clinical outcomes can be examined using a distributed data collection and analysis software tool. This tool applied survival analysis to real-world data drawn from the EHRs of multiple general practices. This exemplar study was able to examine the relationship of statin use in older patients with all-cause mortality in general practice patients aged 75 years and over.

Our methodological approach produced both grouped outcomes but also individual practice-level reports. These reports provide important analysis and feedback which are efficient and appropriate for clinicians to review performance and measure outcomes. The Medical Board of Australia has recently mandated performance review and outcome measurement as a core component of continuing education and professional development, citing international experience, and a large body of evidence to support this approach. (The Australian Health Practitioner Regulation Agency, 2021)

In the future, such a model as demonstrated here may be able to use a meta-analytical approach using these practice-level reports, thereby obviating the need for any patient-level data to leave the practice environment. With further development, research using a distributed data collection and analysis tool maybe an important component of a learning health system, where valid, up-to-date examination of outcomes continually informs future improvements in care.

In addition to the practice-level reports, this study was able to produce research outcomes relevant to primary care. Our study demonstrated that older diabetics who were not prescribed statins had higher mortality rates than those who were. However, we were unable to demonstrate a statistically significant survival advantage for other subgroups of older patients being prescribed statins.

Patients older than 75 who have had their statin prescriptions discontinued had lower rates of all-cause mortality than those who continued to be prescribed statins. This survival advantage was not present for all subgroups, including males, diabetics, and patients treated as primary prevention. However, there was no evidence that cessation of statin prescriptions was associated with increased risk of all-cause mortality in these subgroups.

Our findings are broadly consistent with those from other studies of statin use in older patients. The seminal clinical trial designed to examine outcomes of statin use in older patients, PROSPER, did not demonstrate a difference in all-cause mortality.(Shepherd *et al.*, 2002) A sub-analysis of patients aged over 70 in the JUPITER trial also demonstrated similar all-cause mortality between statin users and non-users. (Glynn *et al.*, 2010) Examination of 726 patients over 75 years of age in a sub-analysis of the ALLHAT-LLT study similarly showed no mortality benefit. (Han *et al.*, 2017)

There have been similar international studies which have used primary care electronic health record data to examine mortality rates associated with statin use in older patients. Ramos et al found that there was a higher risk of mortality in type II diabetics who were not using statins compared to those who were, but otherwise statins did not demonstrate a survival advantage for older patients. (Ramos *et al.*, 2018) While our research replicated the findings of this Spanish study, British research using survival analysis with EHR data described results at odds with our findings. Gitsels *et al.,* (2021) found a survival benefit in all older patients prescribed statins. Awad *et al.,* (2021) performed a meta-analysis of observational studies and concluded that statins provided a survival benefit when used as primary prevention in older patients. However, the survival benefit was significant only in older people with diabetes and not in those without diabetes. (Awad *et al.*, 2021) Further research with a greater representation of Australian general practice data and with a detailed analysis approximating large international studies is highly desirable. We have demonstrated that this is feasible using the approach described here.

It is important to realise the limitations of this research. This exemplar study does not investigate the reasons behind GPs’ decisions to prescribe or to not prescribe a statin medication, nor does it consider the measured lipid levels in the patients. The classification of statin use is based on intention to treat however there remains a possibility that due to prescriptions obtained elsewhere, some patients may have been misclassified. The duration of statin treatment was not quantified. Compliance is not assessed in this study, and it is possible that patients who were prescribed statins did not actually take them. Ethnicity was not assessed as a confounder in this study.

The practices involved in the study were all located in regional NSW. Although a wider geographic spread and variation in rural and metropolitan practices may have improved the generalisability of the results, this was not the aim of the study. Rather it was intended to demonstrate the feasibility of the methodological approach, including the ability to scale the data collection. The limitation introduced by the narrow diversity of practice characteristics is a result of the study being designed as an exemplar of the methodological approach rather than a definitive, large-scale, generalisable research project.

The large number of patients assessed is a strength of the study. The data were recorded contemporaneously in the EHR and are likely representative of Australian regional general practice activity. (Henderson *et al.*, 2018) The results are based on real-world data and are drawn from the spectrum of general practice patients, many of whom would be excluded from clinical trials.

Our study demonstrates no significant survival advantage for older patients prescribed statins (with the exception of diabetics). This carries significant utility for clinicians evaluating treatment requirements for this age group. We can conclude that general practice patients over the age of 75 who were prescribed statins but then received no further prescriptions for at least a year or more (stoppers) were not more likely to die than patients who had their statin scripts continued. In fact, there was a reduced mortality rate among this cohort. Correlation is not causation. The reasons for this outcome may be complex and are beyond the scope of this research. However, it may reassure clinicians and their patients that deprescribing statin medication has not resulted in increased mortality among their older patients.

## Conclusion

This study demonstrates a valuable methodological approach to collect real-world data for concurrent practice-level analysis and generalised, valid, relevant research outcomes. The concordance of our study with existing research supports the finding that with the exception of diabetics, statins may not improve all-cause mortality in older patients. It also provides new evidence that the deprescribing decisions of Australian GPs in relation to statins in older patients do not appear to result in increased mortality.
